# Association lupus systémique et VIH/sida à propos de neuf observations au Centre Hospitalier Universitaire de Libreville (Gabon)

**DOI:** 10.48327/mtsi.v4i4.2024.594

**Published:** 2024-11-05

**Authors:** Josaphat IBA BA, Stéphanie NTSAME NGOUA, Ingrid NSENG NSENG ONDO, Annick Flore MFOUMOU, Jean Bruno BOGUIKOUMA

**Affiliations:** 1 Service de médecine interne, CHU de Libreville, BP 2228, Libreville, Gabon; 2 Service de dermatologie, CHU de Libreville, BP 2228, Libreville, Gabon; 3 Service de rhumatologie, CHU de Libreville, BP 2228, Libreville, Gabon

**Keywords:** VIH1/sida, Lupus systémique, HAART, CD4, Syndrome de restauration immunitaire systémique (SRIS), Gabon, Afrique subsaharienne, HIV1/AIDS, Systemic lupus, HAART, CD4, Systemic immune restoration syndrom (SIRS), Gabon, Sub-Saharan Africa

## Abstract

**Introduction:**

L'infection par le VIH/sida sévit à l’état d'endémie en Afrique subsaharienne. Elle est révélée par des affections opportunistes diverses qui apparaissent habituellement pour un taux de CD4 inférieur à 200/mm^3^, mais également au cours de syndrome de restauration immunitaire (SRI) survenant sous traitement antirétroviral hautement actif *(Highly active antiretroviral therapy,* HAART). Les maladies auto-immunes et inflammatoires sont rarement associées à cette affection. Nous rapportons neuf observations gabonaises d'association lupus systémique-VIH.

**Matériel et méthodes:**

Il s'agit d'une étude rétrospective, descriptive et analytique menée du 1^er^ juin 2016 au 30 avril 2024, dans le service de médecine interne du CHU de Libreville, recensant tous les patients présentant cette association pour en préciser les différentes caractéristiques.

**Résultats:**

Neuf patientes de 36 ans d’âge moyen ont été retrouvées. Le lupus systémique était concomitant au diagnostic d'affection à VIH1 (n = 1) ou survenait après initiation du traitement antirétroviral (n = 6), ou d'une nouvelle ligne d'antirétroviral (n = 2) avec un taux de CD4 moyen lors du diagnostic de 284,5/mm^3^, passant à 578,3/mm^3^.

**Discussion et conclusion:**

Il existe une similitude structurale à l'origine de production d'autoanticorps au cours du VIH1 et du lupus systémique avec des signes cliniques, biologiques, et immunologiques parfois superposables qui peuvent rendre le diagnostic de cette association difficile.

## Abstract (en)

**Background:**

HIV/AIDS infection is endemic in sub-Saharan Africa. It is manifested by a variety of opportunistic diseases that usually occur when the CD4 count is below 200/mm^3^, but also during the immune restoration syndrome (IRS) that occurs under highly active antiretroviral therapy (HAART). Autoimmune and inflammatory diseases are rarely associated with this condition. We report nine Gabonese cases of systemic lupus associated with HIV.

**Material and methods:**

This was a retrospective, descriptive and analytical study conducted from 1 June 2016 to 30 April 2024 at the Department of Internal Medicine of the CHU of Libreville. All patients with this association were identified to specify the different characteristics

**Results:**

Nine female patients with an average age of 36 years were identified. Systemic lupus was associated with the diagnosis of HIV1 disease (n = 1) or occurred after the initiation of antiretroviral treatment (n = 6) or a new line of antiretroviral treatment (n = 2), with a mean CD4 count at diagnosis of 284.5/mm^3^, rising to 578.3/mm^3^.

**Discussion and conclusion:**

There is a structural similarity at the origin of autoantibody production in HIV1 and systemic lupus, with clinical, biological and immunological signs that are sometimes superimposed, which may make the diagnosis of this association difficult.

## Introduction

L'infection par le VIH/sida et les maladies auto-immunes induisent un dysfonctionnement immunitaire dont les mécanismes sont variables, conduisant *in fine* à un tableau d'immunosuppression. Dans le cas de l'infection par le VIH/sida, le VIH va infiltrer un lymphocyte CD4 et y inclure son matériel génétique favorisant la réplication du virus [8]. Au cours des maladies auto-immunes, il existe un dysfonctionnement du système immunitaire conduisant à une perte de reconnaissance des différentes structures de l'organisme avec pour conséquence un tableau d'immunodépression [3]. Au cours de l'infection par le VIH/sida, les maladies auto-immunes peuvent être diagnostiquées concomitamment ou apparaître lors de syndrome de restauration immunitaire (ensemble des manifestations pathologiques liées à une réaction inflammatoire paradoxale en réponse à une infection infraclinique, ou antérieurement traitée, ayant lieu pendant la phase de reprise de fonction du système immunitaire suite au traitement antirétroviral) [16, 18]. Cette coexistence infection par le VIH et maladies auto-immunes reste peu mentionnée dans la littérature médicale, particulièrement en Afrique subsaharienne. Nous rapportons neuf observations gabonaises d'association VIH-lupus.

## Matériel et méthode

Notre étude rétrospective et descriptive a été réalisée dans le service de médecine interne du CHU de Libreville du 1^er^ juin 2016 au 30 avril 2024. Nous avons recensé les dossiers de patients VIH+ dont le diagnostic de lupus systémique (LS) était concomitant ou secondairement diagnostiqué sur la base des critères de l’American College of Rheumatology (ACR) de 1997, suivis en hospitalisation dans ce service, et/ou en consultation. La présence d'au moins 4 des 11 critères de l’ACR permet d'affirmer l'existence d'un LS avec une sensibilité et une spécificité de 96 % [1].

Les variables socio-démographiques de l’étude portaient sur l’âge, le sexe et le statut social. Concernant l'infection par VIH, le taux de CD4 initial ou lors du diagnostic de cette association, la/les affection(s) opportuniste(s) lors ou précédant le diagnostic, le traitement antirétroviral initié (première ou seconde ligne) étaient précisés. Lorsque le LS était diagnostiqué, son délai de survenue suivant la mise sous traitement antirétroviral, le type d'atteinte, les données immunologiques et le traitement instauré étaient précisés. Pour l'analyse, les données quantitatives étaient décrites à l'aide de moyennes, et les données qualitatives à l'aide d'effectifs et de pourcentages.

## Résultats

Neuf patientes VIH1 ont été incluses dans l’étude. Le statut social était majoritairement sans emploi (n = 5), puis fonctionnaire (n = 3), et élève (n = 1). L’âge moyen des patientes était de 36 ans (extrêmes 19 et 48, intervalle de confiance à 95 % (IC) [14,2-42,3]), et 7 patientes sur 9 avaient plus de 30 ans. Le LS était diagnostiqué après la découverte de l'infection VIH1 dans 8 cas, concomitant du diagnostic dans 1 cas, et après l'initiation d'un traitement VIH1 de première (n = 5), ou de seconde ligne (n = 3). Les caractéristiques des patientes sont détaillées dans les Tableaux I et II. Les atteintes du LS étaient plus fréquemment a) cliniques : articulaire (n = 7 patients), musculaire (n = 5), cutanée (n = 3), et cardiaque (n = 2 patients); b) immunologiques : anticorps antinucléaires (n = 8), de spécificité anti-ADN (n = 4), et c) répondaient favorablement à une corticothérapie orale de 1 mg/kg/jour à base de prednisone (précédée par un déparasitage par albendazole : 2 prises de 400 mg/jour pendant 3 jours afin d’éviter une anguillulose disséminée favorisée par l'immunodépression des corticoïdes) couplé à de l'hydroxychloroquine 400 mg/jour, sans survenue d'autre affection opportuniste. L'infection VIH1 était découverte à un taux moyen de CD4 de 284,5/mm^3^ (extrêmes : 6 et 584, IC [169,4-399,6]), passant à 566,6/mm^3^ (extrêmes 350 et 900, IC [427,1-706,1]) après traitement antirétroviral hautement actif (HAART) détaillé dans le Tableau III. Le HAART associé à une corticothérapie orale à base de prednisone (1 mg/kg/jour) et d'hydroxychloroquine (400 mg/jour) s'accompagnait d'une régression des signes cliniques, et d'une augmentation du taux de CD4 dans tous les cas où le dosage a été réalisé.

**Tableau I:** Profil biologique et thérapeutique des patientes lupiques gabonaises du 01/06/2016 au 30/04/2024

N°	Âge	Sexe	Statut social	CD4	Affections opportunistes	TTT ARV	Initié
1	19 ans	Femme	Sans emploi rémunéré	325/mm^3^		1^re^ ligne	TDF/ 3TC/ DTG
2	29 ans	Femme	Sans emploi rémunéré	125/mm^3^		2^e^ ligne	TDF/3TC/ DTG
3	33 ans	Femme	Sans emploi rémunéré	228/mm^3^		2^e^ ligne	TDF/3TC/ DTG
4	34 ans	Femme	Laborantine	383/mm^3^		1^re^ ligne	TDF/3TC/ DTG
5	36 ans	Femme	Élève	584/mm^3^	Tuberculose pulmonaire	1^re^ ligne	ABC/3TC / AZT
6	43 ans	Femme	Sans emploi rémunéré	6/mm^3^		1^re^ ligne	ABC/3TC / AZT
7	47 ans	Femme	Infirmière	245/mm^3^		2^e^ ligne/ 2^nd^ linee	TDF/3TC/ DTG
8	48 ans	Femme	Sans emploi rémunéré	CD4 positif CV 9720000(log 6,99)		1^re^ ligne	
9	35 ans	Femme	Fonctionnaire	380/mm^3^		1^re^ ligne	ABC/3TC / AZT

Légende/Legend : TTT ARV : Traitement antirétroviral/ART: Antiretroviral therapy.

TDF : ténofovir/tenofovir 3TC : lamivudine/lamivudine DTG : dolutégravir/dolutegravir ABC : abacavir/abacavir AZT : zidovudine/zidovudine.

**Tableau II:** Circonstances de diagnostic du lupus des patientes lupiques gabonaises du 01/06/2016 au 30/04/2024

N°	Délai SRIS	Signes cliniques	Données AAN	Immunologiques anticorps anti-ADN	CD4 lors du SRIS
1	1 mois	Vespertilio, polyarthralgie, myalgies, lésions muqueuses	320 moucheté	14 IU/ml	544/mm^3^
2	6 ans	Purpura diffus, Syndrome de Raynaud, vascularite paume des mains, Arthrite distale des mains, myalgies	sup 1 280, moucheté	175 IU/ml	600/mm^3^
3	2 ans	Photosensibilité, alopécie, lésions muqueuses, érythème des paupières, polyarthralgie, myalgies	NR	Test de Coombs+	422/mm^3^
4	7 mois	Hidradénite axillaire bilatérale, alopécie, myalgies	1 280 moucheté	170 IU/ml	NR/NP (383/mm^3^)
5	Concomitant VIH	Péricardite	Positif	43 IU/ml	584/mm^3^
6	9 ans	Polyarthralgie	320 moucheté	Négatif	350/mm^3^
7	4 ans	Polyarthralgie, myalgies, lésions muqueuses	320 moucheté	Négatif	900/mm^3^
8	1 mois	Vespertilio, polyarthralgie	sup 1 280, moucheté	Négatif	NR/NP (CD4 + CV/VL 9720000(log 6,99))
9	1 mois	Vespertilio, polyarthralgie, myalgies, péricardite	sup 1 280, moucheté	71 IU/l	NR (380/mm^3^)

Legende/Legend : AAN : anticorps antinucleaires/ANA: antinuclear antibodies. NR : non realise/NP: not performed. CV : charge virale/VL: viral load

**Tableau III:** Évolution du taux de CD4 lors du SIRS des patients lupiques gabonais du 01/06/2016 au 30/04/2024

N°	Taux CD4	Type d’ARV	Type de ligne	Taux CD4
1	325	TDF/ 3TC / DTG	1^re^ ligne	544
2	125	TDF/ 3TC / DTG	2^e^ ligne	600
3	228	TDF/ 3TC / DTG	2^e^ ligne	422
4	383	TDF/ 3TC / DTG	1^re^ ligne	NR
5	584	ABC/3TC /AZT	1^re^ ligne	Concomitant
6	6	ABC/3TC /AZT	1^re^ ligne	350
7	245	TDF/ 3TC / DTG	2^e^ ligne	900
8	18	TDF/ 3TC / DTG	1^re^ ligne	NR
9	380	ABC/3TC /AZT	1^re^ ligne	NR

Légende/Legend : ABC : abacavir; 3TC : lamivudine; AZT : zidovudine; ARV : traitement antirétroviral; TDF : ténofovir; DTG : dolutégravir ABC: abacavir. 3TC: lamivudine. AZT: zidovudine. ART: Antiretroviral therapy. TDF: tenofovir. DTG: dolutégravir

La tuberculose pulmonaire était inaugurale de la découverte de l'infection VIH1 dans un cas, et une altération de l’état général était présente dans sept cas.

## Discussion

Le LS est une maladie rare, dont la prévalence dans la population générale est inférieure à 50/100 000 habitants. Il affecterait plus fréquemment les femmes en activité génitale avec un sex-ratio de neuf femmes pour un homme. Cette maladie serait 2 à 5 fois plus fréquente et plus sévère chez les sujets noirs ou d'ascendance noire du fait d'une susceptibilité génétique plus marquée et reconnue pour les maladies auto-immunes [17]. L'infection par le VIH peut être associée à des maladies autoimmunes mais également favoriser l'apparition d'autoanticorps isolés sans traduction clinique [11]. Cette production d'autoanticorps au cours de l'infection par le VIH est expliquée d'une part par des similarités structurelles entre les divers épitopes du virus (notamment la protéine p24) et les cellules de l'hôte à l'origine de la production d'autoanticorps [15] et, d'autre part, par une stimulation antigénique persistante et incontrôlée des lymphocytes B mémoire qui entraîne une production soutenue d'anticorps [2].

La première observation de cette association VIH1-LS a été rapportée par Kopelman et Zolla-Pazner en 1988 [6]. Elle peut être différemment intégrée selon que le VIH ou le LS est la pathologie inaugurale. Lorsque le LS est inaugural (aucun cas dans notre série), la contamination par le VIH est rare. Pour Sekigawa *et al*.[13], les patients atteints de LS auraient des niveaux élevés d'interleukine 16, qui, outre son rôle d'indicateur fiable de l'activité de la maladie lupique [7], inhiberait le VIH *in vitro* et représenterait une protection possible contre le VIH chez les patients lupiques. Lorsque l'infection à VIH préexiste ou est inaugurale, l'apparition du LS peut s’établir dans le cadre d'un syndrome de restauration immunitaire systémique (SRIS), peu recherché par les praticiens au cours de l'infection à VIH. Dans une méta-analyse, Liao *et al.* retrouvaient 34/76 patients qui développaient un LS avant le VIH1 *versus* 21/76 un LS après la survenue du VIH/sida, et 11/76 dont le diagnostic des deux affections était concomitant [9].

L'ensemble des caractéristiques de nos patients est compatible avec un SRIS. Les critères diagnostiques de ce syndrome ont été établis par Shelburne *et al.* [14]. Ils retiennent : a) l'apparition de manifestations cliniques après l'introduction d'un traitement antirétroviral efficace (diminution de l’ARN VIH<1 log copies/ml), b) une augmentation habituelle des CD4, mais non constante, c) des manifestations inflammatoires atypiques et d) des manifestations non expliquées par une infection nouvellement acquise, un échec du traitement d'une infection préalablement identifiée (résistance, inobservance) ou un effet indésirable du traitement. Le diagnostic de SRIS peut être retenu chez nos patients, même si dans les SRIS précoces (concomitant et survenant à 1 mois dans notre étude) nous n'avons pas pu refaire un dosage de CD4.

Si dans le traitement de l'anguillulose, le *gold standard* demeure l'ivermectine, le coût élevé de cette médication dans notre pays, (27 € contre 14,4 € pour une cure complète d'albendazole), nous a fait recourir à l'albendazole à la dose de 800 mg/jour pendant 3 jours en prévention d'une anguillulose maligne [4]. Cette possibilité de doubler les doses ne figure pas dans les indications du fabricant, ce qui justifie d'utiliser plutôt l'ivermectine dont l'efficacité apparaît supérieure [10].

En Afrique subsaharienne, qui cumule le plus grand nombre de patients VIH, les observations de cette association demeurent parcellaires. Cela est probablement dû à la difficulté du diagnostic du LS au cours de cette association, du fait de la similarité des signes cliniques (fièvre, arthralgie, lésions muqueuses, syndrome sec), biologiques (cytopénies et hypergammaglobulinémie) [19], et immunologiques (anticorps antinucléaires, et anticorps antiphospholipides) avec l'infection à VIH-1) [6]. Toutefois, les lésions spécifiques aiguës (vespertilio) et chroniques (lupus discoïde) du LS peuvent conduire les praticiens à doser des autoanticorps spécifiques (anti DNA, anti Sm). Dans le cas de la patiente n°5 avec péricardite lupique, il n'existait pas d'argument biologique et paraclinique, même si la tuberculose sévit à l’état d'endémie en Afrique subsaharienne. En outre, la mise sous traitement spécifique du LS s'est accompagnée de la résolution de tous les signes cliniques et biologiques sans récidive.

L'existence d'une positivité des autoanticorps antinucléaires n'est pas suffisante pour le diagnostic de LS. En effet, 5 à 20 % de la population générale possèderaient des autoanticorps antinucléaires à un titre faible sans signes cliniques, avec pour certains la possibilité de ne jamais développer de lupus [12]. Pour être retenue, cette positivité des autoanticorps antinucléaires doit être nécessairement corrélée à des signes cliniques.

Bien que l'incidence varie considérablement dans la littérature médicale, pour Filippidis *et al.* [5], la majorité des patients VIH+ développe au moins une manifestation auto-immune au cours de leur vie.

## Conclusion

Le LS n'est pas une affection opportuniste du sida, mais une manifestation du syndrome de restauration immunitaire peu recherchée par les praticiens au cours de l'infection à VIH. La similitude des signes cliniques, biologiques, et immunologiques, fait de cette association un véritable défi pour les cliniciens.

## Financement de l’étude

L’étude n'a bénéficié d'aucun financement.

## Consentement des patientes

Nous avons obtenu le consentement des patientes pour la publication de cet article.

## Contributions des auteurs et autrices

Iba Ba Josaphat : conception et rédaction; Ntsame Ngoua Stéphanie : rédaction et relecture; Nseng Nseng Ondo Ingrid : relecture et collecte des données; Mfoumou Annick Flore : recherche bibliographique, relecture; Boguikouma Jean Bruno : conception, relecture, approbation de la version finale

## Conflits d'intérêts

Les auteurs et autrices ne déclarent aucun conflit d'intérêt.
