# Supplementary material for: Association of systemic lupus and HIV/AIDS: nine observations at the Centre Hospitalier Universitaire de Libreville (Gabon)
Source: Med Trop Sante Int. 2024 Nov 5;4(4):mtsi.v4i4.2024.594. [Article in French] doi: 10.48327/mtsi.v4i4.2024.594 (PMC11892384; doi:10.48327/mtsi.v4i4.2024.594)
Supplement: Related texts

# Research article: Association of systemic lupus and HIV/AIDS: nine observations at the Centre Hospitalier Universitaire de Libreville (Gabon)

## Introduction

HIV/AIDS infection and autoimmune diseases induce immune dysfunction by various mechanisms, ultimately leading to immunosuppression. In HIV/AIDS infection, HIV infiltrates a CD4 lymphocyte and incorporates its genetic material, which promotes viral replication [8]. In autoimmune diseases, the immune system malfunctions, leading to a loss of recognition of the body's various structures, resulting in immunodepression [3]. In the course of HIV/AIDS infection, autoimmune diseases may be diagnosed concomitantly or occur during the immune restoration syndrome (a set of pathological manifestations related to a paradoxical inflammatory response in response to a subclinical or previously treated infection, occurring during the phase of restoration of immune system function following antiretroviral treatment) [16, 18]. The coexistence of HIV infection and autoimmune disease is rarely reported in the medical literature, especially in sub-Saharan Africa. We report nine cases of HIV-lupus association in Gabon.

## Materials and methods

Our retrospective and descriptive study was conducted in the Department of Internal Medicine, University Hospital of Libreville, from 1 June 2016 to 30 April 2024. We reviewed the records of HIV+ patients with a concomitant or secondary diagnosis of systemic lupus (SL), based on the 1997 American College of Rheumatology (ACR) criteria, who were followed up in hospital in this department and/or in consultation. The presence of at least 4 of the 11 ACR criteria confirms the presence of SL with a sensitivity and specificity of 96% [1]. Sociodemographic variables in the study included age, gender and social status. For HIV infection, initial CD4 count or CD4 count at diagnosis, opportunistic disease(s) at or before diagnosis, and antiretroviral treatment (first or second line) were reported. If diagnosed, time from antiretroviral treatment initiation to onset of the SL, type of disease, immunological data and treatment initiated were reported. For analysis, quantitative data were described as mean values and qualitative data as numbers and percentages.

## Results

Nine HIV1 female patients were included in the study. Social status was predominantly unemployed (n = 5), followed by civil servants (n = 3) and students (n = 1). The mean age of the patients was 36 years (extremes 19 and 48 years, 95% confidence interval (CI) [14.2-42.3]), and 7 out of 9 were older than 30 years. SL was diagnosed after detection of HIV1 infection in 8 cases, concomitantly with diagnosis in 1 case, and after initiation of first-line (n = 5) or second-line (n = 3) HIV1 treatment. Patient characteristics are detailed in Tables I and II. LS involvement was most common a) clinically: joint (n = 7 patients), muscle (n = 5), skin (n = 3) and heart (n = 2); b) immunologically: antinuclear antibodies (n = 8), specific anti-DNA antibodies (n = 4), and c) responded favourably to oral prednisone-based corticosteroid therapy at 1 mg/kg/day (preceded by deworming with albendazole: 2 doses of 400 mg/day for 3 days to avoid disseminated anguillulosis favored by corticosteroid immunosuppression) in combination with hydroxychloroquine 400 mg/day, without the occurrence of other opportunistic diseases. HIV-1 infection was detected at a mean CD4 count of 284.5/mm^3^ (range 6 to 584, CI [169.4-399.6]), which increased to 566.6/mm^3^ (range 350 to 900, CI [427.1-706.1]) after highly active antiretroviral therapy (HAART), as shown in Table III. HAART combined with oral corticosteroids based on prednisone (1 mg/kg/day) and hydroxychloroquine (400 mg/day) was associated with a regression of clinical symptoms and an increase in CD4 count in all cases where the test was performed.

**Table I:** Biological and therapeutic clinical profile of Gabonese lupus patients from 01/06/2016 to 30/04/2024

No	Age	Sex	Social status	CD4	Opportunistic infections	TTT ART	Initiated
1	19 years	Female	Unpaid employment	325/mm^3^		1^st^ line	TDF/ 3TC/ DTG
2	29 years	Female	Unpaid employment	125/mm^3^		2^nd^ line	TDF/3TC/ DTG
3	33 years	Female	Unpaid employment	228/mm^3^		2^nd^ line	TDF/3TC/ DTG
4	34 years	Female	Laboratory assistant	383/mm^3^		1^st^ line	TDF/3TC/ DTG
5	36 years	Female	Student	584/mm^3^	Pulmonary tuberculosis	1^st^ line	ABC/3TC / AZT
6	43 years	Female	Unpaid employment	6/mm^3^		1^st^ line	ABC/3TC / AZT
7	47 years	Female	Nurse	245/mm^3^		2^nd^ linee	TDF/3TC/ DTG
8	48 years	Female	Unpaid employment	CD4 positif CV 9720000(log 6,99)		1^st^ line	
9	35 years	Female	Civil servant	380/mm^3^		1^st^ line	ABC/3TC / AZT

Légende/Legend : TTT ARV : Traitement antirétroviral/ART: Antiretroviral therapy.

TDF : ténofovir/tenofovir 3TC : lamivudine/lamivudine DTG : dolutégravir/dolutegravir ABC : abacavir/abacavir AZT : zidovudine/zidovudine.

**Table II:** Lupus diagnosis circumstances of Gabonese patients from 01/06/2016 to 30/04/2024

No	SIRS leadtime	Clinical signs	ANA data	Immunological antiDNA antibodies	CD4 during SIRS
1	1 month	Vespertilio, polyarthralgia, myalgias, mucosal lesions	320 speckled	14 IU/ml	544/mm^3^
2	6 years	Diffuse purpura, Raynaud's syndrome, vasculitis palms, arthritis distal hands, myalgias	over 1.280 speckled	175 IU/ml	600/mm^3^
3	2 years	Photosensitivity, alopecia, mucosal lesions, erythema of the eyelids, polyarthralgia, myalgias	NP	Coombs test+	422/mm^3^
4	7 month	Bilateral axillary hidradenitis, alopecia, myalgias	1.280 speckled	170 IU/ml	NP (383/mm^3^)
5	HIV concomitant	Pericarditis	Positive	43 IU/ml	584/mm^3^
6	9 years	Polyarthralgia	320 speckled	Negative	350/mm^3^
7	4 years	Polyarthralgia, myalgias, mucosal lesions	320 speckled	Negative	900/mm^3^
8	1 month	Vespertilio, polyarthralgia	over 1.280 speckled	Negative	NP (CD4 + CV/VL 9720000(log 6,99))
9	1 month	Vespertilio, polyarthralgia, myalgias, pericarditis	over 1.280 speckled	71 IU/l	NP (380/mm^3^)

Legende/Legend : AAN : anticorps antinucleaires/ANA: antinuclear antibodies. NR : non realise/NP: not performed. CV : charge virale/VL: viral load

**Table III:** Evolution of the CD4 rate during the SIRS of Gabonese lupus patients from 01/06/2016 to 30/04/2024

No	CD4 rate	Type of ART	Type of line	CD4 rate
1	325	TDF/ 3TC / DTG	1^st^ line	544
2	125	TDF/ 3TC / DTG	2^nd^ line	600
3	228	TDF/ 3TC / DTG	2^nd^ line	422
4	383	TDF/ 3TC / DTG	1^st^ line	NR
5	584	ABC/3TC /AZT	1^st^ line	Concomitant
6	6	ABC/3TC /AZT	1^st^ line	350
7	245	TDF/ 3TC / DTG	2^nd^ line	900
8	18	TDF/ 3TC / DTG	1^st^ line	NR
9	380	ABC/3TC /AZT	1^st^ line	NR

Légende/Legend : ABC : abacavir; 3TC : lamivudine; AZT : zidovudine; ARV : traitement antirétroviral; TDF : ténofovir; DTG : dolutégravir ABC: abacavir. 3TC: lamivudine. AZT: zidovudine. ART: Antiretroviral therapy. TDF: tenofovir. DTG: dolutégravir

Pulmonary tuberculosis led to the discovery of HIV1 infection in one case, and deterioration in general condition was present in seven cases.

## Discussion

SL is a rare disease with a prevalence of less than 50/100,000 in the general population. It is thought to be more common in sexually active females, with a sex ratio of nine females to one-male. The disease is thought to be 2-5 times more common and severe in blacks or people of black descent, due to a greater genetic susceptibility to autoimmune disease [17]. HIV infection may be associated with autoimmune disease, but it may also favour the appearance of isolated autoantibodies without clinical symptoms [11]. This production of autoantibodies during HIV infection is explained by structural similarities between the various epitopes of the virus (in particular the p24 protein) and the host cells at the origin of autoantibody production [15], and by a persistent and uncontrolled antigenic stimulation of memory B lymphocytes leading to sustained antibody production [2].

The first observation of this HIV1-LS association was reported by Kopelman and Zolla-Pazner in 1988 [6]. It may be incorporated differently depending on whether HIV or SL is the initial pathology. When SL is the initial pathology (no cases in our series), HIV infection is rare. According to Sekigawa *et al.* [13], patients with SL have high levels of interleukin 16, which is not only a reliable indicator of lupus disease activity [7], but also inhibits HIV in vitro and represents a possible protection against HIV in lupus patients. In pre-existing or newly acquired HIV infection, the onset of SL may be noted as part of a systemic immune restoration syndrome (SIRS), which is rarely evaluated by physicians in the course of HIV infection. In a meta-analysis, Liao *et al.* found 34/76 patients who developed SL before HIV1 versus 21/76 who developed SL after the onset of HIV/AIDS, and 11/76 whose diagnosis of both conditions was concurrent [9]. All the features of our patients are compatible with SIRS. The diagnostic criteria for this syndrome were established by Shelburne *et al.* [14]. They include: a) the onset of clinical manifestations after the initiation of effective antiretroviral treatment (decrease in HIV RNA >1 log copies/ml), b) an usual but not steady increase in CD4 count, c) atypical inflammatory manifestations, and d) manifestations that cannot be explained by a newly acquired infection, treatment failure for a previously identified infection (resistance, noncompliance), or an adverse effect of treatment. The diagnosis of SIRS can be maintained in our patients, even though in early SIRS (concomitant and occurring at 1 month in our study) we were unable to repeat a CD4 assay.

Although ivermectin remains the gold standard in the treatment of anguillulosis, the high cost of this drug in our country (27 € compared to 14.4 € for a full course of albendazole) led us to use albendazole at a dose of 800 mg/day for 3 days to prevent malignant anguillulosis [4]. However, the possibility of doubling the dose is not mentioned in the manufacturer's leaflet, justifying the use of ivermectin instead, which appears to be more effective [10].

In sub-Saharan Africa, which has the largest number of HIV patients, observations of this combination remain patchy. This is probably due to the difficulty of diagnosing SL in this association, given the similarity of clinical (fever, arthralgia, mucosal lesions, dry syndrome), biological (cytopenias and hypergammaglobulinemia) [19] and immunological (antinuclear and antiphospholipid antibodies) signs with HIV-1 infection [6]. However, the specific acute (vespertilio) and chronic (discoid lupus) lesions of SL may lead clinicians to test for specific autoantibodies (anti-DNA, anti-Sm). In the case of patient n°5 with lupus pericarditis, there were no biological or paraclinical arguments, although tuberculosis is endemic in sub-Saharan Africa. In addition, the specific treatment of SL was associated with resolution of all clinical and biological signs without recurrence.

The presence of positive antinuclear autoantibodies is not sufficient to diagnose SL. Actually, 5-20% of the general population are thought to have low titers of antinuclear autoantibodies without clinical signs, and some may never develop lupus [12]. For antinuclear autoantibody positivity to be accepted, it must be correlated with clinical signs. Although the incidence varies widely in the medical literature, according to Filippidis *et al* [5] the majority of HIV+ patients will develop at least one autoimmune manifestation during their lifetime.

## Conclusion

SL is not an opportunistic disease associated with AIDS, but a manifestation of the immune restoration syndrome, which is little sought after by physicians in the course of HIV infection. The similarity of clinical, biological and immunological signs makes this association a real challenge for clinicians.

## Study funding

The study did not receive any funding.

## Patients’ consent

The patients’ consent was obtained for the publication of this article.

## Authors’ contributions

Iba Ba Josaphat: design and writing; Ntsame Ngoua Stéphanie: writing and proof-reading; Nseng Nseng Ondo Ingrid: proofreading and data collection; Mfoumou Annick Flore: bibliographical research, proofreading; Boguikouma Jean Bruno: design, proofreading, approval of the final version

## Conflicts of interest

The authors declare no competing interests.
